# Changes in work behavior during pregnancy in rural Anhui, China from 2001–03 to 2009: a population based cross-sectional study

**DOI:** 10.1186/s12905-016-0313-7

**Published:** 2016-07-08

**Authors:** Subas Neupane, Bright I. Nwaru, Zhuochun Wu, Elina Hemminki

**Affiliations:** School of Health Sciences, FI-33014 UNiversity of Tampere, Tampere, Finland; Centre for Medical Informatics, Usher Institute of Population Health Sciences and Informatics, The University of Edinburgh, Edinburgh, UK; School of Public Health, Fudan University, Shanghai, China; National Institute for Health and Welfare, Helsinki, Finland

**Keywords:** Pregnancy, Working patterns, Maternal work, Rural China

## Abstract

**Background:**

In low- and middle-income countries, many women continue working later into pregnancy. In our recent study on some areas in rural China, most women stopped working already during the first trimester (≤3 months) of pregnancy. In this paper we aimed to explore whether stopping work during early pregnancy has changed over an 8 year period (between 2001–03 and 2009); we also studied whether the reasons for stopping work early were the same in the two time periods.

**Methods:**

A population-based cross-sectional survey with a representative sample of new mothers was carried out in one rural county in Anhui Province in 2001–03 (*N* = 1479 respondents) and in two other rural counties in 2009 (*N* = 1574 respondents). Both surveys were used to evaluate prenatal care interventions not related to work behavior. The surveys targeted all women who had recently given birth. Multilevel logistic regression analysis was used to examine the determinants of work behavior in the two time periods.

**Results:**

There was a big change in the working behavior between the two survey years: in the period 2001–03 6 % and in 2009, 53 % of pregnant women stopped working at ≤3 months (percentage change 839, 95 % CI −15.90 to 1694.49). In 2001–03, 30 % and in 2009, 23 % of pregnant women worked the same as before pregnancy (percentage change −22.30, 95 % CI −90.28 to 45.68). In both time periods women with two children were less likely to stop work at ≤3 months of pregnancy. Non-farmers were more likely in 2001–03 but less likely in 2009 to stop work at ≤3 months of pregnancy. Women with medium township-level income were more likely to maintain the same level of work as before pregnancy in 2001–03, while in 2009 women with high township-level income were less likely to work the same.

**Conclusion:**

Stopping work very early during pregnancy appeared to have become very common from 2001–3 to 2009 in rural Anhui, China and was not explained by women’s background characteristics.

## Background

Work during pregnancy is not in and of itself a risk, even though some work can be a threat to maternal health and fetal outcomes [1–3]. Maternal work during pregnancy, especially high work-related exertion, physical work demands, long working hours, shift work and occupational stress are also still considered as the most prevalent risk factors of negative pregnancy outcome [4–10].

Although several studies have been published on women’s work and pregnancy outcomes, only few of these have focused on women’s working behavior during pregnancy in low- and middle-income countries [11–13]. In China, in the late 1980s and early 1990s, paid work involved full-time work of 8 h a day, six days a week [14]. However, agricultural fieldwork was more flexible than salaried work, but for most women, the work was equally or more demanding. Nowadays the world of work has changed in rural China; especially women’s work during pregnancy has become more flexible, including shorter working hours, additional breaks, or reduction in the work required.

In a survey in 2009 in three rural provinces in China [13], we found that most women in two of the provinces stopped work already during the first trimester (≤3 months) of pregnancy. In this paper, using the opportunity provided by a survey undertaken in an earlier period (2001–03) in one of these provinces, we wanted to find out whether stopping work early in pregnancy was an old or a new phenomenon. We were also interested in ascertaining the determinants of work behavior and whether these were the same in the two time periods.

## Methods

### Study design and subjects

This study is based on two population-based surveys conducted in 2001–03 and 2009 in rural areas of Anhui Province, China. The surveys have been described in detail elsewhere [15, 16] and only the key features are given here. The first study was conducted as a prenatal care Knowledge, Attitude and Practice (KAP) survey involving 20 rural townships in one county in 2001–2003. The KAP survey was used to evaluate a prenatal care intervention carried out in 1999 [15, 17]. In 2001, interviews were conducted in 10 % of the villages, in 2002 in 20 % and in 2003 in 70 %. Participants in the survey were women who had given birth during the preceding 12 months and who had been identified from a list provided by the family planning system. At the time, family planning system kept records of all life events, including pregnancies and births. Overall, 1479 women (97 % response rate) completed the survey; 3 % of the sample was missing due to refusal, being absent, or for other reasons. Women whose children had died were not interviewed. The non-respondents were few because only few were working out of town or refused to participate in the interview. Proxy respondents (a husband or another family member) responded if the mother was not at home. Health workers from the townships trained by a local researcher employed by Fudan University carried out the survey.

The second survey was conducted in 2008–2009 as a tool to evaluate prenatal care interventions implemented in rural areas of three provinces (Anhui, Chongquing and Shaan’xi) [16–18]. Thirty townships in two counties (not the same as in 2001–2003) of Anhui Province were selected for the intervention or to serve as controls. Participants in the survey were the women who had given birth in the study counties during the year 2008 [18, 19]. Women to be interviewed were identified through various methods: through doctors and family planning workers, the birth registers of township hospitals and snowballing. A letter of invitation explaining the purpose of the study was given or sent to the women [18]. Altogether 1576 women were interviewed (estimated response rate 73 %). Proxy respondents i.e., relatives of the women (7 % of the study population) [20] were used if the respondents were not available at home in the second attempt. Non-participants were working out of town, had an unclear address, were not at home (with no proxy respondents available), or had transportation problems. Trained interviewers conducted the interview.

The interview instrument was a 60-item structured questionnaire in the first survey and a 79-item questionnaire in the second survey. In both surveys, data on mothers’ demographics, work behavior, the content of prenatal care and other information on maternal health care and health outcomes were collected. The first survey instrument was used as a model for the second instrument. The questions on work behavior were identical in the two surveys and the other questions used in this paper were comparable. Questions on income varied (see below), but a comparable classification was used. The questionnaires in both surveys were developed in English, translated into Chinese, and checked against the English version by a bilingual researcher.

### Variables used

The socio-demographic covariates studied in this study were maternal age (≤24 years, 25–29 years and ≥30 years), mother’s income during the first study in 2001–03 (low = <209 $, medium = 209–229 $, high= >229 $ (1 US dollar was equivalent of 8.29 CNY at the time of the study in 2001), while in 2009 total family income was collected instead of mother’s own income (<2174 $, 2174–4348 $, >4348 $: 1 US dollar was equivalent of 6.9 CNY at the time of the study in 2009), number of children at the time of interview (one, two, three), mother’s occupation (farmer, non-farmer). Township-level income was calculated by aggregating mothers’ incomes in 2001–03 and aggregating family total incomes in 2009 and dividing them into three categories (low, medium, and high) based on their tertile values. Prenatal visits during the pregnancy included visits to the doctor/midwife at the township hospital; their visits to her; and mothers’ visits to other health practitioners because of pregnancy. The time of starting prenatal care was categorized into ≤3 months, 4–5 months, 6 months, 7–9 months’ gestation, and no care.

### Work during pregnancy

Work outside home during pregnancy was measured with the question: ‘which of the following statements best describes your working outside home (farming work in the fields or in paid work) during pregnancy?’ with the following responses (a) I worked the same as before pregnancy; (b) I worked less heavily than before pregnancy from … months; and (c) I stopped working completely from … months. For analyses, we created the following categories; worked the same, stopped work at ≤3 months, stopped work >3 months, decreased work at ≤3 months, decreased work >3 months. If a woman had chosen many alternatives, she was grouped in the order given above [6]. Thus, the option ‘stopped work’ may also include reducing work in another stage of pregnancy.

### Statistical analysis

Descriptive statistics were calculated and presented as numbers and percentages. The percentage changes in work behavior and their 95 % confidence intervals (CI) were calculated. The statistical significances of the differences in the proportions were tested using Pearson’s chi-square test. To calculate the associations between maternal characteristics and working behavior, multilevel logistic regression was used. A multilevel modeling procedure was employed [21] as the structure of the data used for analyses was individuals nested within villages and villages nested in townships. In the model, all individual level characteristics (maternal age, time of start of prenatal care, parity, mother’s occupation, mother’s income/family total income) and township level income were simultaneously adjusted. The random part of the models was estimated by computing the variance of the township-level variations and their accompanying standard errors. All analyses were performed using generalized linear latent and mixed models in stata 11‘gllamm’, (Stata Corp LP, College Station, TX).

## Results

Table 1 shows the background characteristics of mothers in 2001–03 and 2009. The age distributions were relatively similar. The proportion of women starting prenatal care visits early increased and the proportion of mothers having three children or more decreased over time. In 2001–03 fewer women (14 %) had a non-farming occupation than in 2009 (27 %). The income level was much higher in 2009 than in 2001–03.

**Table 1:** Background characteristics of women participating in the surveys in 2001–03 and 2009 in Anhui Province, China

Characteristics	2001–03		2009	
	(*N* = 1479)		(*N* = 1574)	
	N	%	N	%
Age				
≤ 24 years	482	32.6	592	37.6
25–29 years	573	38.7	567	36.1
≥ 30 year	374	25.3	415	26.3
No information	50	3.4	-	-
Time of starting prenatal care				
3 months or less	618	41.8	1005	63.5
4–5 months	459	31.0	391	14.8
6 months	177	12.0	84	5.3
7–9 months	90	6.1	47	3.0
No care^a^	135	9.1	47	3.0
Number of children				
1	905	61.2	1078	68.5
2	338	22.9	487	30.9
3	236	16.0	9	0.6
Occupation				
Farmer	1220	82.5	1147	72.9
Non-farmer	209	14.1	427	27.1
No information	50	3.4	-	-
Women’s income^b^ (Mean, SD)	217.0 (212.3)	-		
Family total income^c^ (Mean, SD)	-	3521.2 (3591.6)		

^a^Includes both no care and non-response

^b^Only women’s income (in US $) was collected in 2001–03

^c^Only family total income (in US $) was collected in 2009

The working behaviors during pregnancy were very different in the two time periods (Table 2): In 2001–03 only 6 % of women had stopped working in early pregnancy (≤3 months), while over half had done so in 2009 (% change 839, 95 % CI −15.90 to 1694.49). On the other hand, the proportion of women who had reduced their workload was smaller in 2009 than in 2001–03 (% change for decreased work ≤ 3 months, −69.4 95 % CI −97.6 to −41.2). The proportion of women working the same as before pregnancy was smaller in 2009 than in 2001–03 (% change −22.3 95 % CI −90.3 to 45.7).

**Table 2:** Distribution (%) of women’s work behavior during pregnancy in 2001–03 and 2009 in rural areas in Anhui Province of China

Working patterns during pregnancy	2001–03	2009	*P*-value^a^	Percentage change in work behavior between 2001–03 and 2009
	(*N* = 1479)	(*N* = 1574)		% change (95 % CI)
	%	%		
Worked the same	30.0	23.3	<0.001	−22.3 (−90.3 to 45.7)
Stopped work ≤ 3 months	5.6	52.6	<0.001	839.3 (−15.9 to 1694.5)
Stopped work > 3 months	4.7	11.5	<0.001	144.7 (−82.5 to 371.9)
Decreased work ≤ 3 months	32.7	10.0	<0.001	−69.4 (−97.6 to −41.2)
Decreased work > 3 months	26.4	2.4	<0.001	−90.9 (−99.1 to −82.8)
Missing	0.6	-		

^a^ *P*-value for the differences in the proportion of work behavior between two time periods

Table 3 presents the adjusted associations between the women’s socio-demographic characteristics and work behavior in 2001–03 and 2009. Women’s age had no statistically significant association with stopping work early in pregnancy in either survey. Women having their second child were less likely than women having their first child to stop working early in pregnancy in both surveys. Women’s income, family total income, and township-level income made no difference to stopping work early in the same way in the two time periods. In 2001–03, farmers were less likely and in 2009 more likely to stop working early. In 2001–03, but not in 2009, women starting prenatal care very late (at 7 months of pregnancy or later) were more likely to stop working early.

**Table 3:** Odds ratios (OR) and 95 % confidence intervals (CI) of determinants for stopping working early in pregnancy and for working the same as before pregnancy in rural areas in Anhui Province, China in 2001–03 and 2009

Variables	Stopped work ≤ 3 months^a^		Worked the same during pregnancy	
	aOR (95 % CI)^d^		aOR (95 % CI)^d^	
	2001–03	2009	2001–03	2009
	*N* = 83	*N* = 829	*N* = 444	*N* = 367
Age				
≤ 24 years	1.0	1.0	1.0	1.0
25–29 years	1.10 (0.66–1.85)	0.95 (0.74–1.23)	**1.70 (1.25–2.30)**	1.06 (0.77–1.46)
≥ 30 year	0.54 (0.22–1.30)	0.72 (0.50–1.05)	**1.71 (1.16–2.52)**	1.30 (0.83–2.03)
Time of starting prenatal care				
3 months or less	1.0	1.0	1.0	1.0
4–5 months	1.62 (0.92–2.84)	0.88 (0.68–1.12)	1.13 (0.82–1.56)	0.98 (0.72–1.33)
6 months	1.07 (0.46–2.45)	0.54 (0.28–1.01)	1.33 (0.87–2.05)	1.75 (0.88–3.48)
7–9 months	**2.94 (1.32–6.54)**	1.00 (0.63–1.60)	1.43 (0.83–2.46)	0.61 (0.32–1.16)
No care	1.38 (0.57–3.33)	0.89 (0.48–1.65)	**2.83 (1.82–4.38)**	1.29 (0.65–2.55)
No. of children				
1	1.0	1.0	1.0	1.0
2	**0.44 (0.20–0.97)**	**0.62 (0.45–0.85)**	1.31 (0.92–1.85)	**1.48 (1.02–2.15)**
3	**0.37 (0.16–0.85)**	^c^	1.06 (0.72–1.56)	^c^
Women’s occupation				
Farmer	1.0	1.0	1.0	1.0
Non-farmer	**2.45 (1.37–4.39)**	**0.72 (0.56–0.91)**	0.67 (0.44–1.02)	**1.79 (1.35–2.37)**
Women’s Income				
Low (<209$)	1.0		1.0	
Medium (209–229$)	1.30 (0.67–2.52)	NA	0.49 (0.22–1.10)	NA
High (>229$)	0.73 (0.35–1.49)	NA	1.66 (0.70–3.94)	NA
Family Total Income				
Low (<2174$)		1.0		1.0
Medium (2174–4348$)	NA	0.99 (0.77–1.29)	NA	0.84 (0.62–1.15)
High (>4348$)	NA	1.12 (0.87–1.45)	NA	0.78 (0.57–1.07)
Township-level Income				
Low	1.0	1.0	1.0	1.0
Medium	0.90 (0.43–1.88)	0.81 (0.55–1.19)	**3.67 (1.55–8.68)**	1.43 (0.77–2.65)
High	0.79 (0.39–1.60)	1.28 (0.85–1.91)	1.55 (0.65–3.68)	**0.49 (0.25–0.98)**
*Model statistics*				
Log likelihood	−287.431	−1048.919	−736.991	−783.90
Township level variance (SE^b^)	0.082 (0.125)	0.110 (0.051)	0.446 (0.176)	0.398 (0.140)

*aOR* odds ratio adjusted simultaneous to the other individual characteristics; *NA* Not available;

^a^Women stopped work ≤ 3 months of pregnancy

^b^ Standard Error

^c^There were only few women having 3 or more children in 2009; thus the odds ratio was not calculated

^d^Statistical significant figures d are marked in bold

Older women were significantly more likely than the youngest women to work the same during pregnancy in 2001–03 but not in 2009 (Table 3). Women with two children were more likely to work the same during pregnancy in both surveys; however, the association was significant only in 2009. Women’s income and family total income were not associated with working the same during pregnancy, but those with medium income were more likely to work the same in 2001–03. Medium township-level income was strongly associated with higher likelihood of working the same during pregnancy in 2001–03, but having high township-level income was related to lesser likelihood of working the same in 2009. Non-farmers were significantly more likely to work the same during pregnancy in 2009 but less likely in 2001–03. Women seeking no prenatal care were significantly more likely to work the same during pregnancy than those who had some prenatal visit in 2001–03 but not in 2009.

## Discussion

This study showed that in rural China in 2001–03 only 6 % of pregnant women stopped working at or before 3 months of pregnancy and that in 2009 more than half of the women stopped working; such findings have not been reported from other settings. The difference between the two time periods suggests that stopping work in early pregnancy is a new phenomenon. The proportion of women who maintain the same level of work as before pregnancy declined somewhat during this period.

In developed countries, pregnant women are often advised to reduce or stop strenuous or dangerous work in order to protect the well-being of the mother and the fetus [22, 23]. Women are not usually advised to stop working completely if they are healthy. Pregnant women doing light work may continue to the last trimester [22].

In our study, the pattern of working behavior appeared not to be substantially explained by women’s socio-demographic characteristics, and only some of the background factors were related to working behavior: maternal age and income were not related to stopping work early; starting prenatal care late increased the likelihood of stopping work early in 2001–3, but not in 2009; being a farmer was related in an opposite way in the two surveys. Women with two or more children were less likely to stop work early. We do not know the reason for the finding, but the following factors may have contributed: having many children may have led to an economic necessity to work, a second-time mother could have been less worried or felt better during pregnancy than a first-time mother. A fourth potential explanation is that the second child may have been unauthorized [24] causing social and economic pressure to work.

What could have caused the large increase in the number of women stopping working in the 2000s? Below we briefly present six potential factors which could have contributed to it and give our subjective estimations of how likely these explanations are: changes in health status, strenuousness of work, pregnancy culture, pregnancy care practices, population policy and income.

Pregnancy produces health changes [25]. We did not have information on the women’s health, but it is unlikely that the average health of pregnant women had declined so radically in such a short time. However, as the study areas were different in the two time periods, health status as a reason cannot be totally excluded.

Most of the women (more than 80 % in 2001–3 and more than 70 % in 2009) were engaged in farming. Farmers were less likely to stop working early in 2001–03 and more likely in 2009. It is not likely that farming had become heavier during that time. However, we do not have information on the division of labor between the woman and her husband. Occupational health and safety in China has improved in the last few years [26], but is not likely to have affected the majority of our study population, who were self-employed farmers.

In China there are many traditional pregnancy and birth practices such as restrictions on diet or activity during pregnancy [27–30], and these vary locally [31, 32]. In traditional Chinese medicine it is believed that maintaining emotional harmony is important because anything that influences a women’s mind or spirit, e.g. too much joy and strenuous physical work, affect the heart and can affect the fetus [33]. However, changes in cultural beliefs are unlikely in such a short time period.

In Chinese prenatal care, various behavioral changes and restrictions are recommended to the mother to protect the unborn child [28]. However, there are no recommendations as regards stopping work early in pregnancy. Women are advised to avoid various activities such as carrying heavy loads or engaging in strenuous work during the first trimester, but there is no advice to stop work completely in the first trimester. In 2001–03 women starting their prenatal care visits late (at 7–9 months of pregnancy) were more likely to stop working early in pregnancy; in 2009 the time of starting prenatal care was not associated with stopping work early. This does not suggest that advice from health care was the cause.

The rules of population policy (one child policy) could have contributed to stopping working. In our study area (Anhui Province), a second child was allowed if the first birth was a daughter [34]. With one or two children allowed, the pregnancy and the future health of the child becomes very important.

The average income increased considerably during the study period. We do not have information on the maternity allowance system or whether women received any compensation if they did not work during pregnancy. However, this was unlikely for farmers, who are self-employed. We think that the most likely reason for the rapid increase in stopping work in early pregnancy was the traditional beliefs about the harmfulness of strenuous physical exercise, the increased value of pregnancy, and the increased wealth making stopping work possible.

### Strengths and limitations

The most important limitation was that the two surveys were done in different counties, even though in the same province. We cannot exclude the possibility, however remote, that the counties were different in respect to working behavior during pregnancy. The surveys had different response rates, but the difference in work behavior was so great that it is unlikely that it could be explained by response rate. We did not have detailed information on the content of these women’s work. However, working the same as before until the end of pregnancy may have meant heavy labor, as most of the women were farmers. Another drawback was that we do not have information on the work content, but only the rough classification into farmers and others. There may have been recall bias in retrospectively assessing women’s patterns of work and start of prenatal care. The women’s income was self-reported, and we do not know how accurate it was.

## Conclusions

Further research is needed on how pregnant women work, by occupation and type of work, and whether work patterns are related to pregnancy outcomes. Country comparisons would be useful to identify local peculiarities.

## Abbreviations

aOR, adjusted odds ratio; CHIMACA, Structural hinders to and Promoters of Good Maternal Care in Rural China; CI, Confidence Intervals; CNY, Chinese Yuan; ICRH, International Center for reproductive Health; KAP, Knowledge, Attitude and Practice
